# Development and validation of predictive models for meige syndrome patients based on oxidative stress markers

**DOI:** 10.3389/fimmu.2025.1536109

**Published:** 2025-05-05

**Authors:** Yingjie Zhu, Runing Fu, Ziang Wang, Xinjie Zhu, Pengbo Feng, Xinyu Feng, Wenping Lian

**Affiliations:** ^1^ Department of Clinical Laboratory, The Third People’s Hospital of Henan Province, Zhengzhou, China; ^2^ Second Clinical Medical Group, Hebei Medical University of Hebei Province, Shijiazhuang, China; ^3^ First Clinical Medical Group, Sanquan College of Xinxiang Medical University, Xinxiang, China

**Keywords:** meige syndrome, oxidative stress marker, nomogram, albumin, gamma-glutamyl transferase, total bilirubin, the urea nitrogen-to-creatinine ratio

## Abstract

**Background:**

Meige syndrome (MS) is a complex neurological disorder with unclear etiology. Accurate prediction of MS risk is essential for facilitating early diagnosis. This study aimed to develop and validate a nomogram for predicting the risk of MS based on oxidative stress markers.

**Methods:**

This retrospective, cross-sectional study included 424 patients with MS and 848 age- and sex-matched healthy controls, with data collected from January 2022 to December 2023. Clinical and laboratory data were extracted from electronic medical records. The MS patients and healthy controls were randomly allocated to the training and validation sets at a 7:3 ratio using random stratified sampling. A nomogram was developed using a multivariate logistic regression model based on data from the training set. Model performance was validated through fivefold cross-validation, receiver operating characteristic (ROC) curves, calibration plots, and decision curve analysis (DCA).

**Results:**

Univariate and multivariate logistic regression analyses identified albumin, gamma-glutamyl transferase (GGT), total bilirubin (TBIL), and the urea nitrogen-to-creatinine ratio as independent predictors of MS. A nomogram was constructed based on these four variables. The cross-validation confirmed the model’s reliability. The model demonstrated high predictive accuracy, with an area under the curve (AUC) of 0.930 for the training set and 0.914 for the validation set. The calibration curve and DCA results indicate that the model has strong consistency and significant potential for clinical application.

**Conclusions:**

This study developed a nomogram based on four risk predictors, GGT, TBIL, albumin, and the urea nitrogen-to-creatinine ratio, to forecast the risk of MS and enhance the accuracy of MS risk prediction.

## Introductions

Meige syndrome (MS), initially reported by the French neurologist Henri Meige in 1910, is an infrequent and debilitating neurological condition characterized by involuntary contractions of the eyelids and facial muscles (1), which ultimately leads to significant functional impairment and psychosocial distress for affected individuals. Generally, the condition is more prevalent among individuals aged 40 to 70, with a higher incidence in women than in men (2). Currently, the etiology and pathogenesis of MS remain unknown. It has been suggested that a disruption in the balance of neurotransmitters in the brain, particularly dopamine and acetylcholine, may be involved (3). This neurochemical imbalance has been associated with multiple factors, such as specific medications, psychological stress, dental interventions, and trauma (4). Diagnosis of MS currently depends largely on patients’ clinical symptoms, and there is no definitive diagnostic test available. Consequently, there is a pressing need to identify reliable biomarkers for MS diagnosis.

Impairment of the basal ganglia and related neural pathways plays a key role in the development of MS (5). Research indicates that MS may be linked to an imbalance between acetylcholine and dopamine, while other studies suggest that changes in neuronal plasticity and decreased cortical inhibition could contribute to the condition (3, 6). A variety of neurological disorders have been linked to neuronal cell death due to oxidative stress (7, 8). Many studies have demonstrated that oxidative stress is a key factor in the progression of various neurological disorders, including Alzheimer’s disease (AD), amyotrophic lateral sclerosis (ALS), Parkinson’s disease (PD), Huntington’s disease (HD), ischemic stroke, and multiple sclerosis (9). The central nervous system (CNS) exhibits heightened sensitivity to oxidative stress, a result of its high metabolic rate, rich presence of substances susceptible to oxidation, and constrained antioxidant capabilities (10). Reactive nitrogen species (RNS) and reactive oxygen species (ROS) reactive can lead to oxidative stress, which inflicts damage on cellular constituents such as lipids, proteins, and DNA, ultimately resulting in cellular impairment and apoptosis (11). Oxidative stress can also modify the activity of ion channels, which are essential for preserving neuronal excitability and signal transmission (12). Oxidative stress-induced alterations in ion channel regulation can exacerbate the progression of neurological diseases (13).

In recent years, biomarkers such as albumin, gamma-glutamyl transferase (GGT), total bilirubin (TBIL), uric acid (UA), and the urea nitrogen-to-creatinine ratio have emerged as significant indicators of oxidative stress in the context of neurological diseases (14–16). Although these markers have been extensively studied in various contexts, their potential as predictors for MS has not been fully explored. The present study innovatively investigates the relationship between these oxidative stress markers and MS, revealing their potential as novel predictive indicators for MS. To the best of our knowledge, this is the first study to explore the association between these oxidative stress markers in blood and MS, with the largest sample size of MS cases reported to date. Given the lack of definitive diagnostic tests for MS and the critical importance of early diagnostic for improved patient outcomes, our nomogram-a powerful predictive tool-provides a quantitative and reliable method for clinicians to assess MS risk based on these oxidative stress markers. This innovation facilitates early intervention and personalized management strategies.

## Materials and methods

### Study population

This retrospective, cross-sectional study included 530 consecutive patients diagnosed with MS who were admitted to the Third People’s Hospital in Henan Province, from January 2022 to December 2023. The study received clearance from the Research Ethics Committee at the Third People’s Hospital of Henan Province (2024-SZSYKY-009) and adhered to the Declaration of Helsinki, as well as the pertinent institutional protocols. Due to the retrospective and observational design of the study, the requirement for informed consent was waived. To safeguard participant privacy, all data extracted from the medical record system were anonymized, ensuring the protection of patient confidentiality.

**Figure 1:**
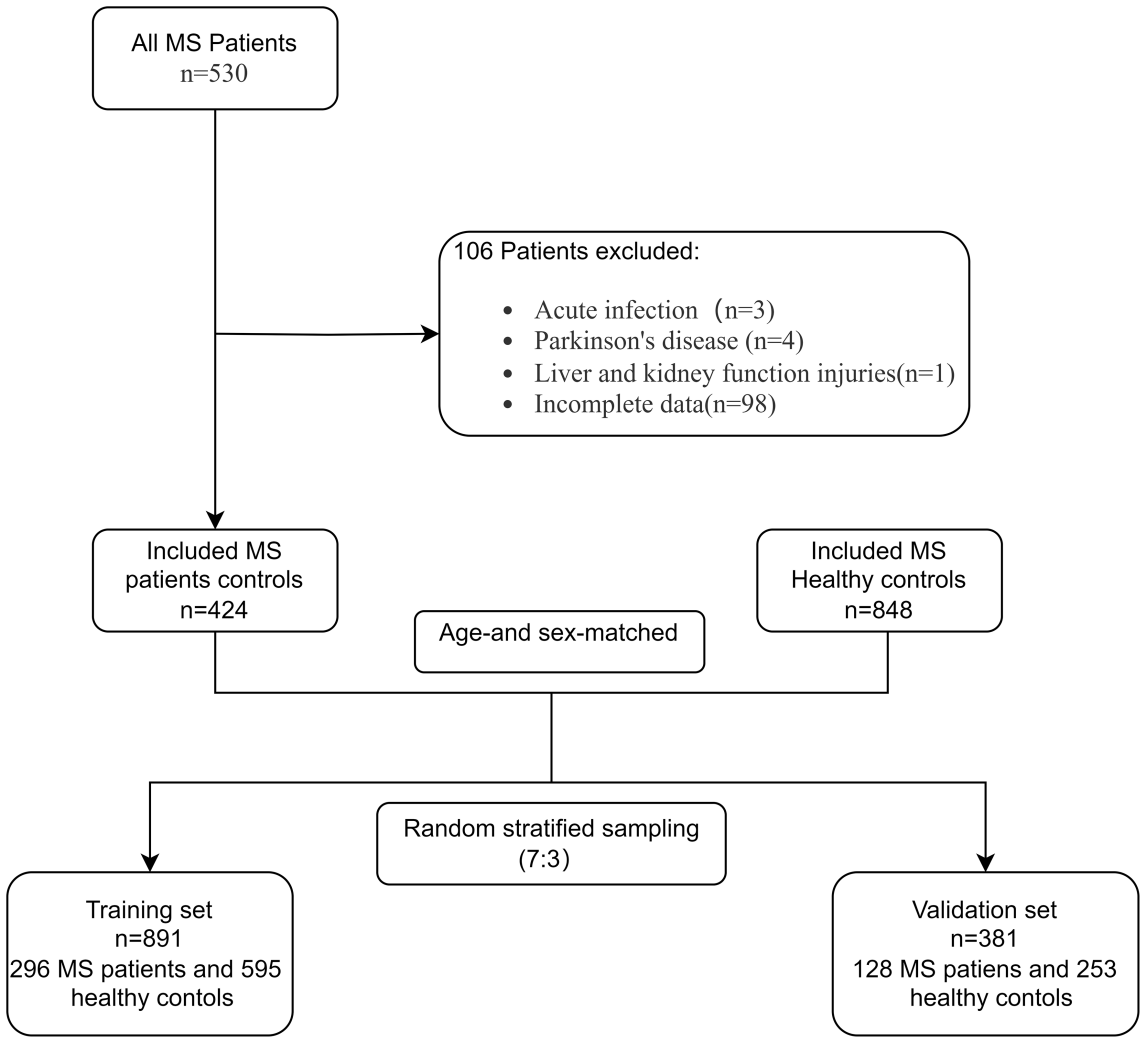
Study flow chart.

Participants were included if they were diagnosed with primary MS by a clinician based on clinical criteria. Exclusions were made for patients with: (1) acute infections, (2) malignant tumors, (3) severe hepatic or renal insufficiency, and (4) incomplete data for key variables such as albumin, GGT, TBIL, indirect bilirubin (IBIL), direct bilirubin (DBIL), total protein, UA, creatinine (CREA), and urea nitrogen (UREA). After applying these exclusions, the study ultimately comprised 424 individuals with MS. Furthermore, data from 848 healthy individuals, matched for age and sex, who had routine medical examinations at the same hospital were gathered for the study. Using a random stratified sampling with a 7:3 ratio, the MS patients and healthy controls were divided into two groups: a training set comprising 296 MS patients and 595 healthy controls, and a validation set consisting of 128 MS patients and 253 healthy controls (**Figure 1**).

### Data extraction

Clinical and laboratory data for all patients were extracted from the electronic medical record system. The clinical data included information on age, sex, and BMI. The laboratory data included GGT (reference range, 7 to 58 U/L), TBIL (reference range, 2 to 20.5 µmol/L), DBIL (reference range, 0.4 to 6.9 µmol/L), IBIL (reference range, 1.7 to 13.2 µmol/L), total protein (reference range, 60 to 85 g/L), albumin (reference range, 35 to 55 g/L), CREA (reference range, 44 to 106 µmol/L), UREA (reference range, 1.7 to 8.2 mmol/L), and UA (reference range, 149 to 369 µmol/L) (Beckman AU680, USA). The urea nitrogen-to-creatinine ratio was calculated as follows: urea nitrogen-to-creatinine ratio = urea nitrogen/creatinine. Data related to epidemiology, clinical assessments, and laboratory tests were collected from each patient’s single visit.

### Statistical analyses

The Shapiro-Wilk test used to evaluate the normality of the data. In cases where the data conformed to a normal distribution, we documented the mean and standard deviation (mean ± SD) and employed the Student’s t-test to discern differences between the two groups. When the data did not follow a normal distribution, we applied the Wilcoxon-Mann-Whitney U test and presented the results as median and interquartile range, denoted as [M (P25, P75)]. For categorical data, comparisons were conducted using Pearson’s chi-square test, and the results were expressed in terms of frequency (percentage). To initially filter variables, a univariate logistic regression analysis was performed, and in combination with the Spearman’s rank correlation coefficient and the variance inflation factor (VIF), predictors for the final model were chosen. Subsequently, the RMS package in R was employed to create nomograms, and the internal validity of the model was confirmed by conducting fivefold cross-validation. The performance of the nomogram was appraised through the analysis of receiver operating characteristic (ROC) curves, calibration plots, and decision curve analysis (DCA). Statistical analyses were performed using SPSS version 25.0, and model construction and validation were conducted using R software (version 4.2.1). The threshold for statistical significance was established at P < 0.05.

## Results

### Patient characteristics

A total of 530 patients with MS were initially screened for eligibility. Of these, 3 patients with infectious diseases, 4 patients with PD, and 1 patient with renal insufficiency impairment were excluded. Additionally, 98 patients with incomplete data were excluded from the analysis. Ultimately, this study included 424 MS patients and 848 healthy controls (HCs) (**Table 1**). There were no significant differences regarding sex, age, and BMI between the groups (P > 0.05). The MS group exhibited higher levels of UREA and the urea nitrogen-to-creatinine ratio compared to HCs (P < 0.05), whereas levels of albumin, total protein, GGT, IBIL, DBIL, TBIL, UA, and CREA were significantly lower in the MS group (P < 0.05). Subsequently, MS patients and HCs were randomly allocated to the training and validation sets at a 7:3 ratio using random stratified sampling. No significant differences were observed between the two groups of MS patients (**Table 2**).

**Table 1:** Characteristics of the patients with MS and healthy controls.

Characteristics	Meige syndrome N=424	Healthy control N=848	P value
Sex			1.000
Male	102 (24.1%)	204 (24.1%)	
Female	322 (75.9%)	644 (75.9%)	
Age	59 (53, 65)	59 (53, 65)	1.000
BMI	24.26 (22.3, 26.318)	24.3 (22.5, 26.6)	0.554
GGT (U/L)	16 (13, 22)	20 (16, 28)	< 0.001
TBIL (µmol/L)	13.7 (11.6, 16.7)	16.15 (13.4, 18.3)	< 0.001
DBIL (µmol/L)	3.7 (3, 4.6)	4.5 (3.6, 5.9)	< 0.001
IBIL (µmol/L)	10 (8.475, 12)	11.5 (9.7, 12.6)	< 0.001
TP (g/L)	67.4 (64.4, 70.4)	74.55 (72.1, 77)	< 0.001
ALB (g/L)	40.6 (38.675, 42.5)	44.8 (43.4, 46.6)	< 0.001
UA (µmol/L)	288.85 (245.1, 331.88)	300.3 (256.6, 346.1)	0.005
CREA (µmol/L)	51.8 (44.5, 60.15)	56.85 (50.375, 65.525)	< 0.001
UREA (mmol/L)	5.23 (4.42, 6.015)	4.835 (4.09, 5.61)	< 0.001
UCR ×100	9.865 (8.25, 12.22)	8.26 (7.11, 9.6425)	< 0.001

Medians and interquartile ranges are utilized to represent continuous variables, while frequencies and percentages are employed to depict categorical variables.

GGT, gamma-glutamyl transferase; TBIL, total bilirubin; DBIL, direct bilirubin; IBIL, indirect bilirubin; TP, total protein; ALB, albumin; UA, uric acid; CREA, creatinine; UREA, urea nitrogen; UCR, urea nitrogen-to-creatinine ratio.

**Table 2:** Characteristics of patients with MS in the training and validation sets.

Characteristics	Training set Meige syndrome N=296	Validation set Meige syndrome N=128	P value
Sex			0.765
Male	70 (23.6%)	32 (25.0%)	
Female	226 (76.4%)	96 (75.0%)	
Age	59 (53, 66)	58 (53, 65)	0.307
BMI	24.26 (22.308, 26.045)	24.28 (22.285, 26.67)	0.679
GGT (U/L)	16 (12.75, 22)	16.5 (13, 21)	0.812
TBIL (µmol/L)	13.6 (11.6, 16.925)	13.85 (11.6, 16.175)	0.854
DBIL (µmol/L)	3.7 (3, 4.7)	3.75 (3, 4.5)	0.892
IBIL (µmol/L)	10.05 (8.4, 12.1)	10 (8.575, 11.8)	0.891
TP (g/L)	66.9 (64.4, 70)	68 (64.4, 70.9)	0.399
ALB (g/L)	40.539 ± 2.5195	40.988 ± 2.8769	0.127
UA (µmol/L)	288.9 (244, 329.9)	285.9 (247.67, 347.27)	0.884
CREA (µmol/L)	51.85 (44.1, 60.65)	51.55 (44.9, 59.225)	0.820
UREA (mmol/L)	5.22 (4.39, 6.0625)	5.28 (4.57, 5.95)	0.414
UCR ×100	9.81 (8.1, 12.287)	10.055 (8.7075, 12.1)	0.484

GGT, gamma-glutamyl transferase; TBIL, total bilirubin; DBIL, direct bilirubin; IBIL, indirect bilirubin; TP, total protein; ALB, albumin; UA, uric acid; CREA, creatinine; UREA, urea nitrogen; UCR, urea nitrogen-to-creatinine ratio.

### Logistic regression analyses in the MS

We conducted a univariate analysis to identify variables with statistically significant differences (P < 0.05). These variables were subsequently incorporated into a multivariate logistic regression analysis to ascertain the independent predictors (P < 0.05) (**Supplementary Figure S1**). To assess multicollinearity, we employed VIF assessment (VIF < 5) and Spearman correlation analysis (r > 0.5) (**Supplementary Table S1**, **Supplementary Figure S2**). After excluding variables with multicollinearity, the multivariate logistic regression identified albumin, GGT, TBIL and urea nitrogen-to-creatinine ratio as independent predictive factors. Specifically, lower levels of albumin (OR, 0.453; 95% CI, 0.410–0.500; P < 0.001), GGT (OR, 0.941; 95% CI, 0.919–0.964; P < 0.001), and TBIL (OR, 0.928; 0.877–0.982; P = 0.009), along with higher levels of the urea nitrogen-to-creatinine ratio (OR, 1.461; 95% CI, 1.352–1.579; P < 0.001), were recognized as independent predictors of disease risk for MS (**Table 3**). Patients with MS were categorized into two groups based on disease duration and whether they had received botulinum toxin injections. The impact of these factors on the four independent variables predicting disease risk for MS was compared. Results indicated that neither disease duration nor botulinum toxin injection status significantly affected the aforementioned indicators (**Supplementary Table S3**, **Supplementary Table S4**).

**Table 3:** Univariate and multivariate logistic regression analyses for MS.

Characteristics	Univariate analysis		Multivariate analysis	
	OR (95% CI)	P value	OR (95% CI)	P value
Male	1.000 (0.761 – 1.314)	1.000		
Age	1.000 (0.987 – 1.014)	1.000		
BMI	0.980 (0.943 – 1.019)	0.317		
ALB (g/L)	0.470 (0.432 – 0.511)	< 0.001	0.453 (0.410 – 0.500)	< 0.001
TP (g/L)	0.628 (0.596 – 0.662)	< 0.001		
GGT (U/L)	0.915 (0.898 – 0.932)	< 0.001	0.941 (0.919 – 0.964)	< 0.001
IBIL (µmol/L)	0.832 (0.788 – 0.879)	< 0.001		
DBIL (µmol/L)	0.642 (0.583 – 0.707)	< 0.001		
TBIL (µmol/L)	0.858 (0.827 – 0.891)	< 0.001	0.928 (0.877 – 0.982)	0.009
UA (µmol/L)	0.997 (0.995 – 0.999)	0.001	1.003 (1.000 – 1.005)	0.058
CREA (µmol/L)	0.951 (0.940 – 0.962)	< 0.001		
UREA (mmol/L)	1.381 (1.244 – 1.533)	< 0.001		
UCR ×100	1.341 (1.274 – 1.412)	< 0.001	1.461 (1.352 – 1.579)	< 0.001

GGT, gamma-glutamyl transferase; TBIL, total bilirubin; DBIL, direct bilirubin; IBIL, indirect bilirubin; TP, total protein; ALB, albumin; UA, uric acid; CREA, creatinine; UREA, urea nitrogen; UCR, urea nitrogen-to-creatinine ratio.

### Construction of the MS-predicting nomogram

**Figure 2:**
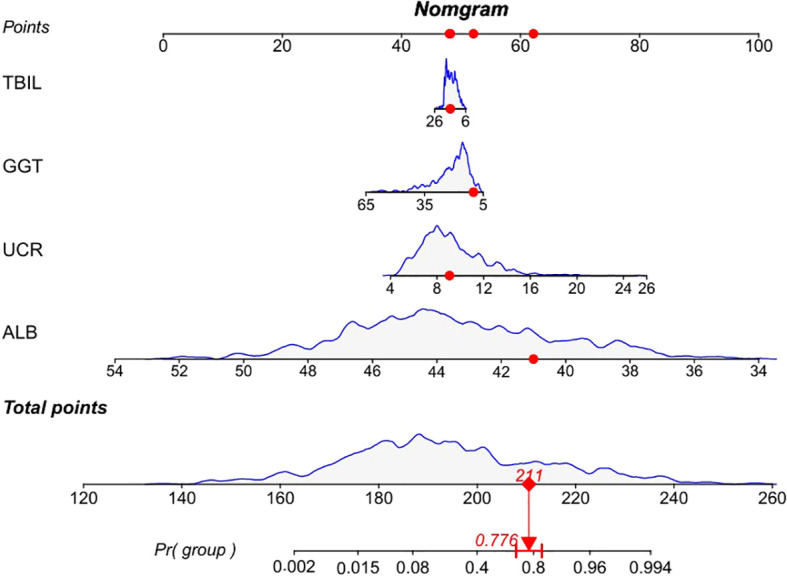
A predictive nomogram for MS. A score is allocated to each variable on a point scale, with the aggregate of these scores yielding the total points. These total points are then mapped to the predicted probability of MS. The red lines represent values of a randomly selected patient from the development dataset who is GGT of 14 U/L (52 points), with TBIL of 11.9 u mol/L (49 point), ALB of 41.1 g/L (62 points), and UCR of 9.92 (48 points). These values when plotted, correspond to the points scale and points are then summed to give a total point score. The presented patient has a total point’s score of 211. The red line indicates that the MS predicted probability corresponding to the total score of this example is 0.776. GGT, gamma-glutamyl transferase; TBIL, total bilirubin; ALB, albumin; UCR, urea nitrogen-to-creatinine ratio.

In the multivariate analysis, four variables with statistically significant differences (P < 0.05), including albumin, GGT, TBIL, and the urea nitrogen-to-creatinine ratio, were used to construct a nomogram for predicting the risk of MS using data from the training set. Based on the nomogram, a comprehensive scoring table was developed, with the total score for the model reaching 260 points. At total score thresholds of 197, 203, and 208 points, the respective predicted probabilities for the development of MS were 30%, 50%, and 70%. In our study, the nomogram was developed using the subsequent formula: prediction probability = 1/(1+exp (0.84464 × albumin + 0.051268 × GGT + 0.040896 × TBIL - 0.3057 × the urea nitrogen-to-creatinine ratio - 34.263)). To illustrate its application, a patient was randomly selected from the dataset for development purposes. The patient had a GGT value of 14 U/L. Additionally, the TBIL value was 11.9 µmol/L, the albumin value was 41.1 g/L, and the urea nitrogen-to-creatinine ratio value was 9.92. From the calculations using the formula, the predicted probability of MS was 0.776 (**Figure 2**). This score offers crucial information for subsequent clinical evaluations and decision-making processes.

### Internal prediction model validation

To validate the reliability and robustness of our predictive model, we conducted internal validation using a fivefold cross-validation method. This approach involves dividing the dataset randomly into five equal subsets. During each round of cross-validation, one of the subsets is designated as the validation set, and the other four subsets are combined to form the training set. This cycle is iterated 100 times, making certain that each data point is utilized once for training and once for validation. The performance of our model was evaluated using the area under the curve (AUC). The AUC values obtained from each of the five folds were averaged to provide a single estimate of the model’s performance. The mean AUC across all folds was 0.93, indicating a high discriminatory power of our model in predicting MS risk based on oxidative stress marker (**Supplementary Figure S3**).

### Validation of the MS-predicting nomogram

**Figure 3:**
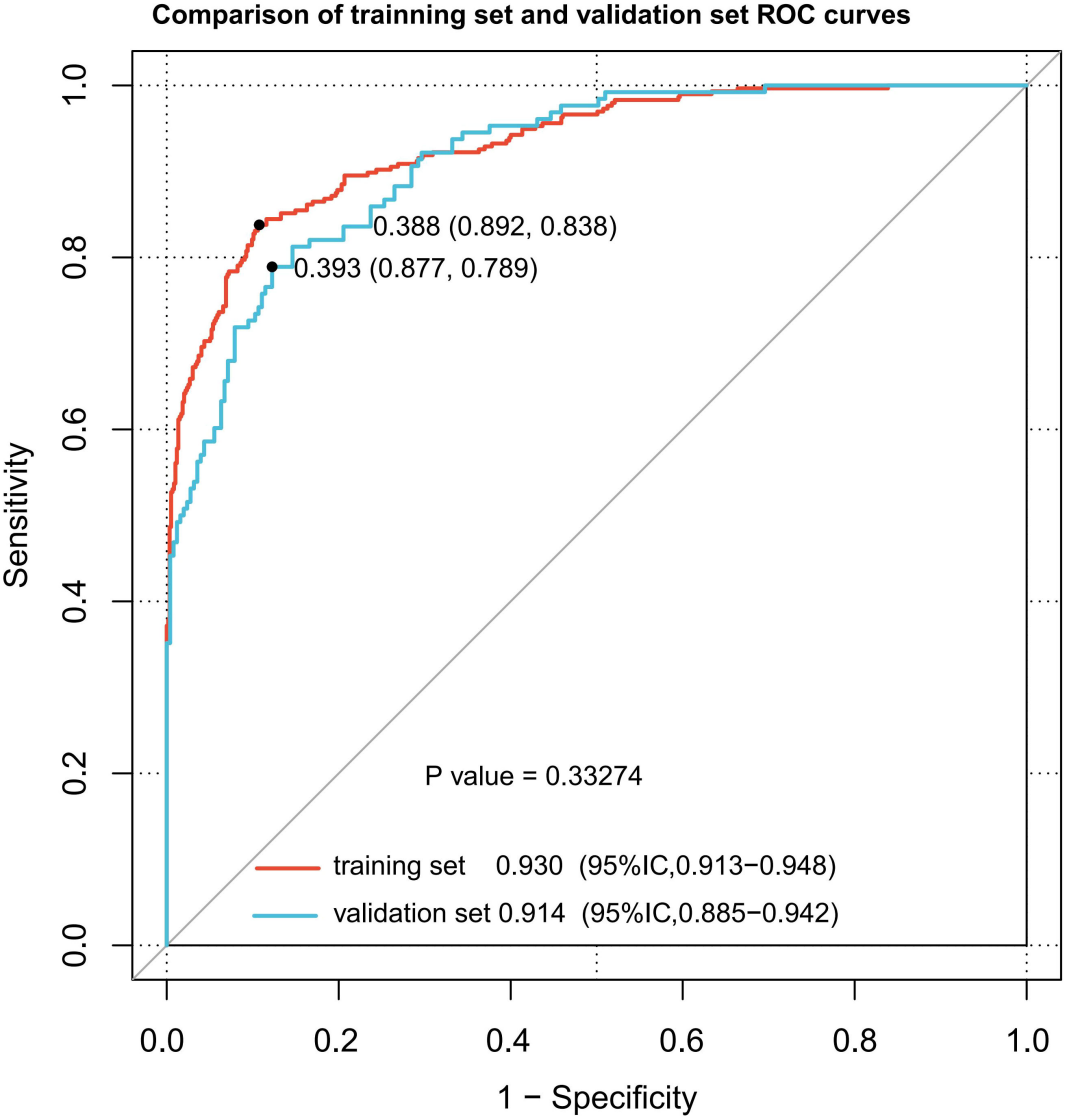
ROC curves for the training and the validation sets. The dot on the curve indicates the point corresponding to the optimal cutoff value.

**Figure 4:**
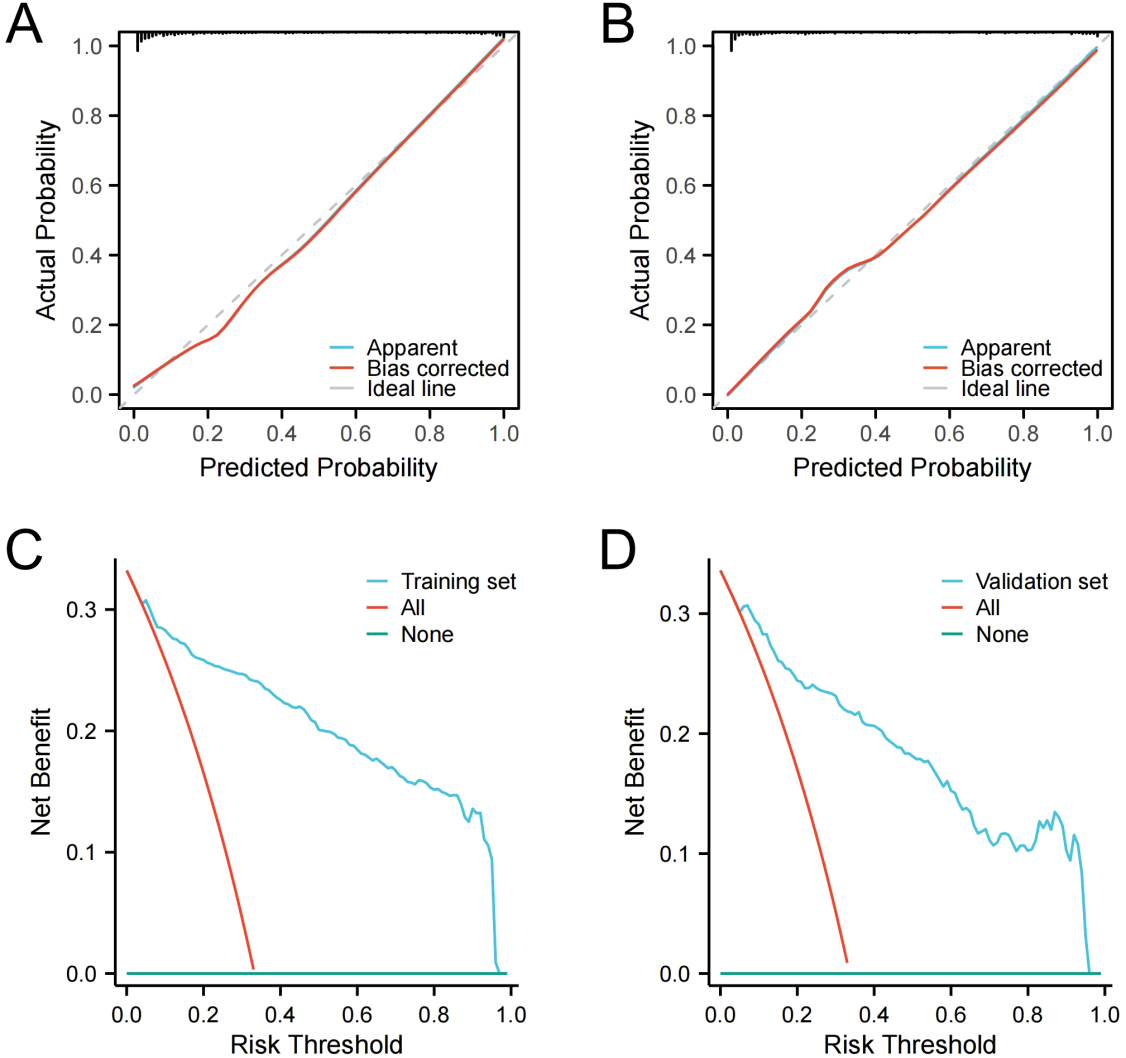
Calibration curve for the training set **(A)**. Calibration curve for the validation set **(B)**. DCA curve for the training set **(C)**. DCA curve for the validation set **(D)**.

The nomogram demonstrated robust discriminative ability across both datasets. For the training set, the nomogram achieved an AUC of 0.930 (95% CI: 0.913–0.948), with a sensitivity of 83.8%, specificity of 89.2%, positive predictive value (PPV) of 84.6%, negative predictive value (NPV) of 88.0%, and a Brier score of 0.092. For the validation set, the AUC was 0.914 (95% CI: 0.885–0.942), with a sensitivity of 78.90%, specificity of 87.70%, PPV of 80.7%, NPV of 86.5%, and a Brier score of 0.110 (**Figure 3**, **Supplementary Table S2**). The calibration curves were constructed to compare the nomogram’s predicted probabilities with actual outcomes in both datasets. Predicted probabilities were categorized into bins, and the mean predicted probability for each bin was plotted against the corresponding observed frequency. The resulting curves closely approximated the 45-degree line, indicating good calibration. Calibration slopes were close to 1 and intercepts were close to 0, further confirming the nomogram’s reliability for outcome prediction (**Figures 4A, B**). Furthermore, DCA was used to evaluate the practical application of the nomogram for predicting MS. With a range of threshold probabilities from 0 to 0.97, the net benefit was positive, indicating the model’s superior clinical value for MS prediction (**Figures 4C, D**). These results indicate that the nomogram possesses robust predictive and discriminative capabilities.

## Discussion

Studies have suggested that oxidative stress plays a significant role in the pathological mechanisms of various neurological disorders (9). Given its high metabolic activity and oxygen demand, the brain is particularly susceptible to oxidative stress. Excessive ROS, including superoxide, hydrogen peroxide, and hydroxyl radicals, can damage vital biomolecules such as proteins, lipids, and DNA (16). This damage initiates a cascade that can result in cellular dysfunction and, ultimately, neurodegeneration. However, current evidence does not link peripheral blood oxidative stress markers to MS. Thus, investigating the relationship between these markers and MS is crucial.

In this study, compared with HCs, the serum levels of antioxidants, such as GGT, TBIL, albumin, and UA were significantly reduced in patients with MS, while the pro-oxidant the urea nitrogen-to-creatinine ratio was markedly increased. This finding lends credence to the hypothesis that oxidative damage could be a significant factor in the development of MS. Multivariate logistic regression analysis identified GGT, TBIL, albumin, and urea nitrogen-to-creatinine ratio as independent risk predictors for MS. These biomarkers are readily accessible and have potential for broad clinical application. We integrated these variables into a nomogram to develop a predictive model. The model demonstrated strong discriminative ability, with ROC curve areas of 0.930 (95% CI: 0.913–0.948) for the training set and 0.914 (95% CI: 0.885–0.942) for the validation set. Calibration curves and DCA indicated that the model has good consistency and clinical utility.

Serum albumin, the most abundant protein in circulation, has antioxidant properties that significantly mitigate reoxygenation injury (17–19). albumin’s antioxidant properties are due to its molecular structure. The reduced Cys34 residue, the largest pool of thiols in circulation, enables albumin to scavenge nitric oxide (NO), ROS, other reactive nitrogen species, and prostaglandins via this cysteine residue (20–22). Albumin exhibits neuroprotective effects, partly due to its modulation of intracellular signaling in neuronal and glial cells and its antioxidant capabilities (23). In recent years, an increasing amount of evidence has indicated that albumin plays a crucial role in neurological diseases. Some studies have linked lower serum albumin levels to a higher risk of cognitive impairment and dementia (24). Furthermore, research has shown that serum albumin levels are notably reduced in patients with PD and are considered independent risk factors for the condition (25). Similarly, we observed that MS patients had significantly lower albumin levels in their peripheral blood compared to HCs. This reduction in albumin implies a weakened antioxidant capacity, which may exacerbate oxidative stress, induce neural tissue damage, and impede neural recovery, thereby potentially promoting MS development. The potential link between albumin levels and MS risk underscores the importance of further investigating albumin as a biomarker for MS diagnosis and risk assessment.

Bilirubin, a potent endogenous antioxidant, is found in biological fluids throughout the body and ranks among the strongest natural antioxidants (25). TBIL is the breakdown product of aging red blood cells (26), exhibits potent antioxidant capabilities, enabling it to neutralize harmful oxidative stress byproducts, shield the body’s organs from injury (27), and has direct antioxidant properties in the brain (28). Under normal conditions, the human body possesses an antioxidant defense mechanism that maintains a dynamic equilibrium between oxidizing agents and antioxidants, thereby preventing harm to the body. However, a disruption in this balance can result in heightened oxidative processes, which can lead to tissue damage (29). Our research indicated that compared to the HCs, the TBIL levels in MS are reduced, aligning with prior studies (15). This reduction may be associated with increased oxidative stress and tissue damage in MS. Additionally, elevated TBIL levels have been reported in patients with Parkinson’s disease (PD), potentially due to dopaminergic substitution therapy (30). Two plausible explanations for the altered TBIL levels in neurological disorders are: First, bilirubin exhibits cytoprotective effects against injury caused by oxidative stress. Second, bilirubin has anti-inflammatory properties. The latter has been particularly evident in brain tissue, as demonstrated in models of experimental autoimmune encephalomyelitis, where bilirubin exhibits potent anti-inflammatory activity (31). Therefore, the deficiency of TBIL in MS patients could exacerbate oxidative stress and inflammation, contributing to disease progression. Further investigation into the relationship between TBIL and MS may provide valuable insights into the pathogenesis of MS and identify TBIL as a potential biomarker or therapeutic target for the disease.

GGT primarily facilitates the “glutathione cycle,” which operates at the plasma membrane and is crucial for maintaining cysteine homeostasis, thereby preserving intracellular glutathione levels and the cell’s redox balance (32). Recent studies indicate that GGT contributes significantly to antioxidant defense and the metabolism of xenobiotics (33). As a key antioxidant, GGT serves as an indicator of oxidative stress, with numerous studies linking its activity to the development of a range of diseases, including cardiovascular disease, cancer, lung inflammation, and neurological disorders (14). In our study, MS patients exhibited lower GGT levels compared to HCs, implying that reduced GGT might be a risk factor for MS. However, this finding differs from the results observed in other neurological disorders. Previous studies have shown that higher GGT levels have been correlated with dementia risk (34). The observed discrepancy may be attributed to the dual nature of GGT, which can promote pro-oxidant activity through the degradation of glutathione in the extracellular space under specific conditions (14). In MS, the reduced GGT levels could indicate a diminished antioxidant capacity, potentially exacerbating oxidative stress and contributing to disease progression.

UREA and CREA are end products of nitrogen-containing compounds in the human body and serve as accessible biomarkers for renal function in clinical settings (35). The urea nitrogen-to-creatinine ratio, a laboratory biomarker, has traditionally been used to assess dehydration (36). Recently, it has also been identified as an independent prognostic factor for adverse outcomes in patients with various conditions, such as stroke (16) and heart failure (37). Other research has determined that a reduced level of the urea nitrogen-to-creatinine ratio could potentially elevate the risk of ischemic stroke (38). However, there is inconsistency in the effects of urea nitrogen-to-creatinine ratio on various diseases, and the role of the urea nitrogen-to-creatinine ratio in MS patients remained uncertain. In MS, the role of the urea nitrogen-to-creatinine ratio was previously unexplored. Our study revealed significantly higher serum the urea nitrogen-to-creatinine ratio levels in MS patients compared to HCs, suggesting its potential as a biomarker for MS susceptibility. Previous studies suggested that urea nitrogen-to-creatinine ratio levels correlate with oxidative stress (39), abnormal inflammation (40), and endothelial dysfunction (41) in patients, which could be potential mechanisms underlying the association between elevated the urea nitrogen-to-creatinine ratio and MS risk. Given the established links between the urea nitrogen-to-creatinine ratio and various adverse outcomes, as well as its association with oxidative stress and inflammation, our findings highlight the potential importance of the urea nitrogen-to-creatinine ratio as a biomarker for MS risk.

This study was the first to build an MS prediction model based on oxidative stress markers. The independent predictors included in our model are easily accessible, thereby enhancing the ease of using the model. However, given that this study is a retrospective, single-center analysis, it also has certain limitations. The single-center origin of the data limits the generalizability of the study results, and the lack of external validation further underscores the need for future studies to employ multicenter data for further verification. Moreover, due to its retrospective nature and the fact that the normal controls were individuals undergoing health check-ups, we were unable to accurately obtain data regarding several potential confounding factors, such as medication history prior to admission and dietary habits.

## Conclusions

Our study successfully developed a nomogram incorporating GGT, TBIL, albumin, and the urea nitrogen-to-creatinine ratio to predict the risk of MS. This model offers a clinically valuable tool for risk assessment, demonstrating robust performance with high AUCs in both training and validation cohorts. The calibration curves and the DCA results demonstrate that the model exhibits strong concordance and possesses significant clinical application value, ultimately highlighting its potential utility in MS predict.

## Data Availability

The raw data supporting the conclusions of this article will be made available by the authors, without undue reservation.

## References

[1] Pandey S Sharma S. Meige’s syndrome: History, epidemiology, clinical features, pathogenesis and treatment. J Neurol Sci. (2017) 372:162–70. doi: 10.1016/j.jns.2016.11.053 28017205

[2] Liu J Li L Li Y Wang Q Liu R Ding H. Metabolic imaging of deep brain stimulation in meige syndrome. Front Aging Neurosci. (2022) 14:848100. doi: 10.3389/fnagi.2022.848100 35370610 PMC8968570

[3] Zeppieri M Ameer MA Jahngir MU Patel BC. Meige syndrome. In: StatPearls. StatPearls Publishing LLC, Treasure Island (FL (2024). ineligible companies. Disclosure: Muhammad Atif Ameer declares no relevant financial relationships with ineligible companies. Disclosure: Muhammad Jahngir declares no relevant financial relationships with ineligible companies. Disclosure: Bhupendra Patel declares no relevant financial relationships with ineligible companies.: StatPearls Publishing Copyright ^©^ 2024.

[4] Arzul L Henoux M Marion F Corre P. Bilateral chronic dislocation of the temporomandibular joints and Meige syndrome. Rev Stomatol Chir Maxillofac Chir Orale. (2015) 116(2):106–10. doi: 10.1016/j.revsto.2015.01.004 25742702

[5] Zheng W Lv G Lu Y Liu J Hao Q Ding H. Bilateral pallidal deep brain stimulation in meige syndrome: effects on motor function, neuropsychological status, and mood. Neurosurgery. (2023) 92:1073–9. doi: 10.1227/neu.0000000000002335 36728352

[6] Ribot B Aupy J Vidailhet M Mazère J Pisani A Bezard E. Dystonia and dopamine: From phenomenology to pathophysiology. Prog Neurobiol. (2019) 182:101678. doi: 10.1016/j.pneurobio.2019.101678 31404592

[7] Jung YS Ryu BR Lee BK Mook-Jung I Kim SU Lee SH. Role for PKC-epsilon in neuronal death induced by oxidative stress. Biochem Biophys Res Commun. (2004) 320:789–94. doi: 10.1016/j.bbrc.2004.05.217 15240117

[8] Elmann A Mordechay S Rindner M Larkov O Elkabetz M Ravid U. Protective effects of the essential oil of salvia fruticosa and its constituents on astrocytic susceptibility to hydrogen peroxide-induced cell death. J Agric Food Chem. (2009) 57:6636–41. doi: 10.1021/jf901162f 19722569

[9] Dash UC Bhol NK Swain SK Samal RR Nayak PK Raina V. Oxidative stress and inflammation in the pathogenesis of neurological disorders: Mechanisms and implications. Acta Pharm Sin B (2025) 15(1):15–34. doi: 10.1016/j.apsb.2024.10.004 40041912 PMC11873663

[10] Adibhatla RM Hatcher JF. Lipid oxidation and peroxidation in CNS health and disease: from molecular mechanisms to therapeutic opportunities. Antioxid Redox Signal. (2010) 12:125–69. doi: 10.1089/ars.2009.2668 19624272

[11] Ashok A Andrabi SS Mansoor S Kuang Y Kwon BK Labhasetwar V. Antioxidant therapy in oxidative stress-induced neurodegenerative diseases: role of nanoparticle-based drug delivery systems in clinical translation. Antioxidants (Basel). (2022) 11(2):408. doi: 10.3390/antiox11020408 35204290 PMC8869281

[12] Orfali R Alwatban AZ Orfali RS Lau L Chea N Alotaibi AM. Oxidative stress and ion channels in neurodegenerative diseases. Front Physiol (2024) 15:1320086. doi: 10.3389/fphys.2024.1320086 38348223 PMC10859863

[13] Bogeski I Niemeyer BA. Redox regulation of ion channels. Antioxid Redox Signal. (2014) 21:859–62. doi: 10.1089/ars.2014.6019 PMC4115641 24930772

[14] Corti A Belcastro E Dominici S Maellaro E Pompella A. The dark side of gamma-glutamyltransferase (GGT): Pathogenic effects of an ‘antioxidant’ enzyme. Free Radic Biol Med. (2020) 160:807–19. doi: 10.1016/j.freeradbiomed.2020.09.005 32916278

[15] Qin XL Zhang QS Sun L Hao MW Hu ZT. Lower serum bilirubin and uric acid concentrations in patients with parkinson’s disease in China. Cell Biochem Biophys. (2015) 72:49–56. doi: 10.1007/s12013-014-0402-x 25449297

[16] Deng L Wang C Qiu S Bian H Wang L Li Y. Association between blood urea nitrogen-to-creatinine ratio and three-month outcome in patients with acute ischemic stroke. Curr Neurovasc Res. (2019) 16:166–72. doi: 10.2174/1567202616666190412123705 30977443

[17] Ferrer R Mateu X Maseda E Yébenes JC Aldecoa C De Haro C. Non-oncotic properties of albumin. A multidisciplinary vision about the implications for critically ill patients. Expert Rev Clin Pharmacol. (2018) 11:125–37. doi: 10.1080/17512433.2018.1412827 29219627

[18] Kremer H Baron-Menguy C Tesse A Gallois Y Mercat A Henrion D. Human serum albumin improves endothelial dysfunction and survival during experimental endotoxemia: concentration-dependent properties. Crit Care Med. (2011) 39:1414–22. doi: 10.1097/CCM.0b013e318211ff6e 21336119

[19] Tokunaga C Bateman RM Boyd J Wang Y Russell JA Walley KR. Albumin resuscitation improves ventricular contractility and myocardial tissue oxygenation in rat endotoxemia. Crit Care Med. (2007) 35:1341–7. doi: 10.1097/01.CCM.0000260242.77637.57 17414087

[20] Anraku M Chuang VT Maruyama T Otagiri M. Redox properties of serum albumin. Biochim Biophys Acta. (2013) 1830:5465–72. doi: 10.1016/j.bbagen.2013.04.036 23644037

[21] Cha MK Kim IH. Glutathione-linked thiol peroxidase activity of human serum albumin: a possible antioxidant role of serum albumin in blood plasma. Biochem Biophys Res Commun. (1996) 222:619–25. doi: 10.1006/bbrc.1996.0793 8670254

[22] Roche M Rondeau P Singh NR Tarnus E Bourdon EJFL. The antioxidant properties of serum albumin. FEBS Letters. (2008) 582:1783–7. doi: 10.1016/j.febslet.2008.04.057 18474236

[23] Prajapati KD Sharma SS Roy N. Current perspectives on potential role of albumin in neuroprotection. Rev Neurosci. (2011) 22:355–63. doi: 10.1515/rns.2011.028 21591907

[24] Wang L Wang F Liu J Zhang Q Lei P. Inverse relationship between baseline serum albumin levels and risk of mild cognitive impairment in elderly: A seven-year retrospective cohort study. Tohoku J Exp Med. (2018) 246:51–7. doi: 10.1620/tjem.246.51 30249938

[25] Wang L Hu W Wang J Fang F Cheng G Jiang Y. Impact of serum uric acid, albumin and their interaction on Parkinson’s disease. Neurol Sci. (2017) 38:331–6. doi: 10.1007/s10072-016-2738-z 27878402

[26] Creeden JF Gordon DM Stec DE Hinds TD Jr. Bilirubin as a metabolic hormone: the physiological relevance of low levels. Am J Physiol Endocrinol Metab. (2021) 320:E191–e207. doi: 10.1152/ajpendo.00405.2020 33284088 PMC8260361

[27] Vitek L Hinds TD Jr. Stec DE Tiribelli C. The physiology of bilirubin: health and disease equilibrium. Trends Mol Med. (2023) 29:315–28. doi: 10.1016/j.molmed.2023.01.007 PMC10023336 36828710

[28] Peng F Yang Y Liu J Jiang Y Zhu C Deng X. Low antioxidant status of serum uric acid, bilirubin and albumin in patients with neuromyelitis optica. Eur J Neurol. (2012) 19:277–83. doi: 10.1111/j.1468-1331.2011.03488.x 21801283

[29] Demirci-Çekiç S Özkan G Avan AN Uzunboy S Çapanoğlu E Apak R. Biomarkers of oxidative stress and antioxidant defense. J Pharm BioMed Anal. (2022) 209:114477. doi: 10.1016/j.jpba.2021.114477 34920302

[30] Scigliano G Girotti F Soliveri P Musicco M Radice D Caraceni T. Increased plasma bilirubin in Parkinson patients on L-dopa: evidence against the free radical hypothesis? Ital J Neurol Sci. (1997) 18:69–72. 9239525 10.1007/BF01999565

[31] Gazzin S Vitek L Watchko J Shapiro SM Tiribelli C. A novel perspective on the biology of bilirubin in health and disease. Trends Mol Med. (2016) 22:758–68. doi: 10.1016/j.molmed.2016.07.004 27515064

[32] Diaz-Vivancos P De Simone A Kiddle G Foyer CH. Glutathione–linking cell proliferation to oxidative stress. J F. R B Med. (2015) 89:1154–64. doi: 10.1016/j.freeradbiomed.2015.09.023 26546102

[33] Brennan PN Dillon JF Tapper EB. Gamma-Glutamyl Transferase (γ-GT) - an old dog with new tricks? Liver Int. (2022) 42:9–15. 34775657 10.1111/liv.15099 PMC11165564

[34] Lee YB Han K Park S Kim SM Kim NH Choi KM. Gamma-glutamyl transferase variability and risk of dementia: A nationwide study. Int J Geriatr Psychiatry. (2020) 35:1105–14. doi: 10.1002/gps.v35.10 32392636

[35] Murata A Kasai T Matsue Y Matsumoto H Yatsu S Kato T. Relationship between blood urea nitrogen-to-creatinine ratio at hospital admission and long-term mortality in patients with acute decompensated heart failure. Heart Vessels. (2018) 33:877–85. doi: 10.1007/s00380-018-1135-3 29417223

[36] Gao B Gu H Yu W Liu S Zhou Q Kang K. Admission dehydration is associated with significantly lower in-hospital mortality after intracerebral hemorrhage. Front Neurol. (2021) 12:637001. doi: 10.3389/fneur.2021.637001 33763017 PMC7982572

[37] Qian H Tang C Yan G. Predictive value of blood urea nitrogen/creatinine ratio in the long-term prognosis of patients with acute myocardial infarction complicated with acute heart failure. Med (Baltimore). (2019) 98:e14845. doi: 10.1097/MD.0000000000014845 PMC6426612 30882678

[38] Peng R Liu K Li W Yuan Y Niu R Zhou L. Blood urea nitrogen, blood urea nitrogen to creatinine ratio and incident stroke: The Dongfeng-Tongji cohort. Atherosclerosis. (2021) 333:1–8. doi: 10.1016/j.atherosclerosis.2021.08.011 34390959

[39] Vaziri ND Dicus M Ho ND Boroujerdi-Rad L Sindhu RK. Oxidative stress and dysregulation of superoxide dismutase and NADPH oxidase in renal insufficiency. Kidney Int. (2003) 63:179–85. doi: 10.1046/j.1523-1755.2003.00702.x 12472781

[40] Giribabu N Karim K Kilari EK Salleh N. Phyllanthus niruri leaves aqueous extract improves kidney functions, ameliorates kidney oxidative stress, inflammation, fibrosis and apoptosis and enhances kidney cell proliferation in adult male rats with diabetes mellitus. J Ethnopharmacol. (2017) 205:123–37. doi: 10.1016/j.jep.2017.05.002 28483637

[41] Chalikias G Tziakas D. Cardiovascular consequences of acute kidney injury. N Engl J Med. (2020) 383:1094. 10.1056/NEJMc2023901 32905694

