# Supplementary material for: Development and validation of predictive models for meige syndrome patients based on oxidative stress markers
Source: Front Immunol. 2025 May 5;16:1536109. doi: 10.3389/fimmu.2025.1536109 (PMC12086067; doi:10.3389/fimmu.2025.1536109)
Supplement: Supplementary file 1 [file DataSheet1.docx]

**Table S1** The variance inflation factor (VIF) assesses multicollinearity, values > 5 indicate potential multicollinearity.

| Variable | VIF |
| --- | --- |
| ALB | 1.378 |
| TP | 1.425 |
| GGT | 1.096 |
| TBIL | 13.563 |
| IBIL | 13.219 |
| UA | 1.306 |
| CREA | 14.248 |
| UREA | 19.200 |
| UCR | 24.986 |

Abbreviations: GGT, gamma-glutamyl transferase; TBIL, total bilirubin; IBIL, indirect bilirubin; TP, total protein; ALB, albumin; UA, uric acid; CREA, creatinine; UREA, urea nitrogen; UCR, urea nitrogen-to-creatinine ratio.


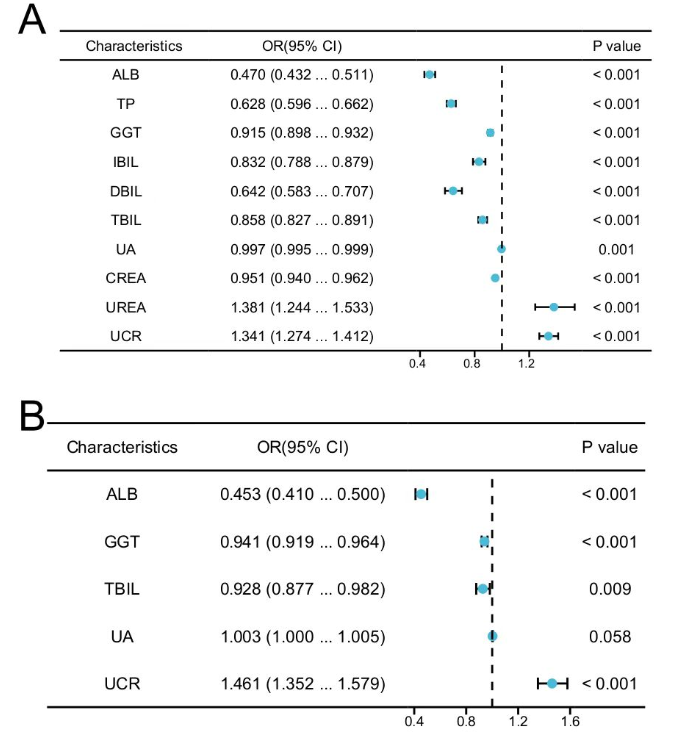


**Figure S1** Forest plots of risk factors for MS with single factor independent prognosis (A) and multifactor independent prognosis (B).

Abbreviations: GGT, gamma-glutamyl transferase; TBIL, total bilirubin; DBIL, direct bilirubin; IBIL, indirect bilirubin; TP, total protein; ALB, albumin; UA, uric acid; CREA, creatinine; UREA, urea nitrogen; UCR, urea nitrogen-to-creatinine ratio.


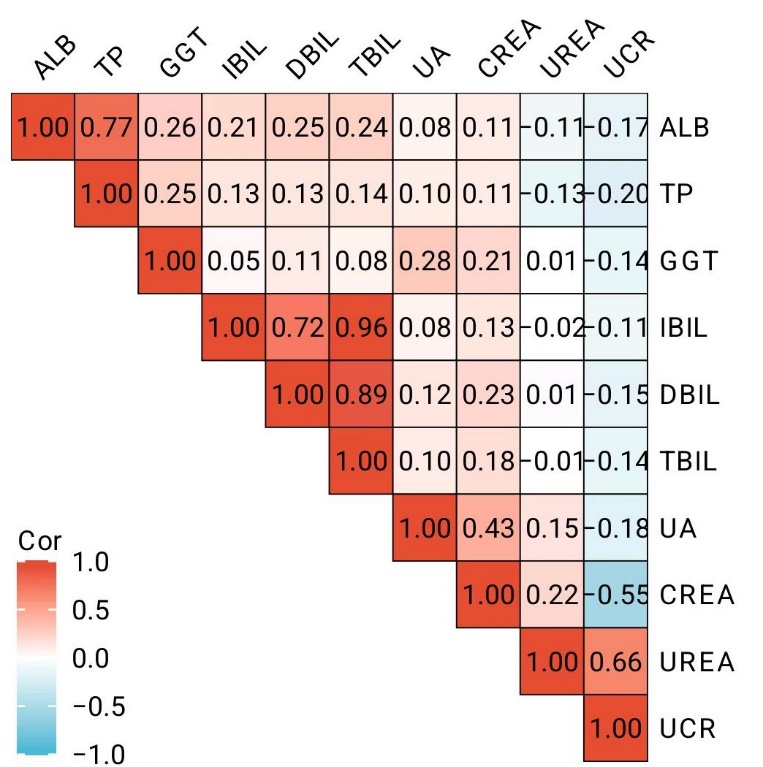


**Figure S2** Heatmap of pairwise correlation coefficients among variables in the dataset.

Abbreviations: GGT, gamma-glutamyl transferase; TBIL, total bilirubin; DBIL, direct bilirubin; IBIL, indirect bilirubin; TP, total protein; ALB, albumin; UA, uric acid; CREA, creatinine; UREA, urea nitrogen; UCR, urea nitrogen-to-creatinine ratio.

**Table S2** Model evaluation metrics.

| Evaluation metrics | Training Set | Testing Set |
| --- | --- | --- |
| PPV | 0.846 (0.802, 0.888) | 0.807 (0.728, 0.882) |
| NPV | 0.880 (0.856, 0.905) | 0.865 (0.823, 0.906) |
| Brier Score | 0.092 (0.079, 0.104) | 0.110 (0.089, 0.130) |

Abbreviations: PPV, positive predictive value; NPV, negative predictive value

**Table S3** Comparison of disease risk predictors in MS patients stratified by disease duration.

| Characteristics | < 4 Years  N=205 | ≥4 Years  N=219 | P value |
| --- | --- | --- | --- |
| ALB (g/L) | 40.4 (38.8, 42.3) | 40.7 (38.4, 43.1) | 0.238 |
| GGT (U/L) | 16 (13, 23) | 16 (12.5, 20) | 0.212 |
| TBIL (u mol/L) | 13.8 (11.5, 16.2) | 13.6 (11.85, 16.95) | 0.345 |
| UCR*100 | 9.58 (8.43, 12.2) | 10.03 (8.115, 12.265) | 0.522 |

Notes: Categorization of MS patients into two groups according to median disease duration: <4 Years and ≥4 Years.

Abbreviations: GGT, gamma-glutamyl transferase; TBIL, total bilirubin; ALB, albumin; UCR, urea nitrogen-to-creatinine ratio.

**Table S4** Comparison of disease risk predictors in MS Patients Stratified by botulinum toxin injection status.

| Characteristics | BTX- injected  N=299 | BTX- not injected  N=125 | P value |
| --- | --- | --- | --- |
| ALB (g/L) | 40.668 ± 2.5349 | 40.616 ± 2.8265 | 0.852 |
| GGT (U/L) | 16 (12.5, 21) | 16 (13, 23) | 0.250 |
| TBIL (u mol/L) | 13.7 (11.65, 16.45) | 13.5 (11.3, 17.5) | 0.983 |
| UCR*100 | 9.66 (8.135, 12.165) | 10.08 (8.28, 12.49) | 0.088 |

Abbreviations: BTX, botulinum toxin; GGT, gamma-glutamyl transferase; TBIL, total bilirubin; ALB, albumin; UCR, urea nitrogen-to-creatinine ratio.

**
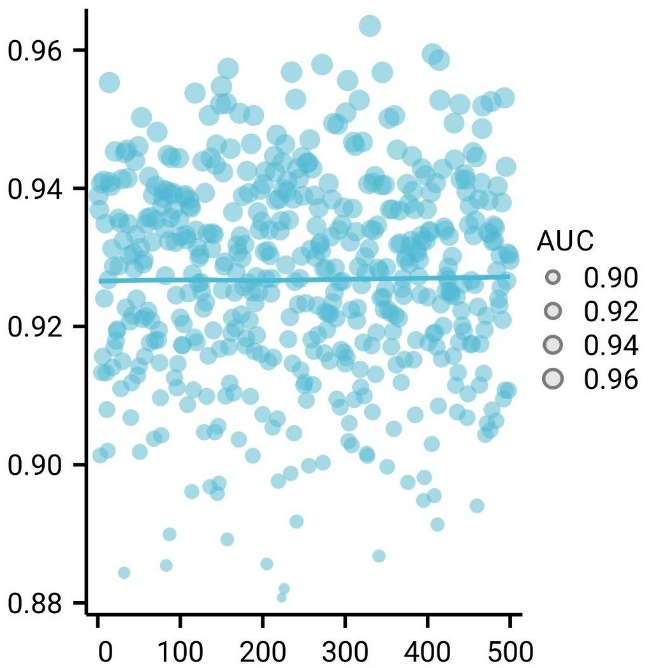
**

**Figure S3** Model validation was conducted via Five-fold cross-validation, with the average AUC serving as the metric for assessment.
